# A patient-derived mouse model reproduces molecular, neurological, and sleep symptoms of SHINE syndrome

**DOI:** 10.1093/hmg/ddag065

**Published:** 2026-08-07

**Authors:** Sharon Tamir, Jiffin Paulose, Anh Nguyen, Darshak Gadara, Rochelle M Witt, Bhagwat Prasad, John B Hogenesch

**Affiliations:** Division of Human Genetics, Department of Pediatrics, Cincinnati Children’s Hospital Medical Center, 3333 Burnet Avenue, Cincinnati, OH 45229, United States; Department of Pediatrics, University of Cincinnati College of Medicine, 3230 Eden Avenue, Cincinnati, OH 45267, United States; Division of Human Genetics, Department of Pediatrics, Cincinnati Children’s Hospital Medical Center, 3333 Burnet Avenue, Cincinnati, OH 45229, United States; Department of Pediatrics, University of Cincinnati College of Medicine, 3230 Eden Avenue, Cincinnati, OH 45267, United States; Division of Human Genetics, Department of Pediatrics, Cincinnati Children’s Hospital Medical Center, 3333 Burnet Avenue, Cincinnati, OH 45229, United States; Department of Pediatrics, University of Cincinnati College of Medicine, 3230 Eden Avenue, Cincinnati, OH 45267, United States; Division of Translational and Clinical Pharmacology, Department of Pediatrics, Cincinnati Children’s Hospital Medical Center, 3333 Burnet Avenue, Cincinnati, OH 45229, United States; Department of Pediatrics, University of Cincinnati College of Medicine, 3230 Eden Avenue, Cincinnati, OH 45267, United States; Division of Neurology and Sleep Center, Department of Pediatrics, Cincinnati Children’s Hospital Medical Center, 3333 Burnet Avenue, Cincinnati, OH 45229, United States; Division of Pulmonary Medicine, Department of Pediatrics, Cincinnati Children’s Hospital Medical Center, Cincinnati, 3333 Burnet Avenue, OH 45229, United States; Center for Circadian Medicine, Cincinnati Children's Hospital Medical Center, 3333 Burnet Avenue, Cincinnati, OH 45229, United States; Department of Pediatrics, University of Cincinnati College of Medicine, 3230 Eden Avenue, Cincinnati, OH 45267, United States; Division of Translational and Clinical Pharmacology, Department of Pediatrics, Cincinnati Children’s Hospital Medical Center, 3333 Burnet Avenue, Cincinnati, OH 45229, United States; Division of Human Genetics, Department of Pediatrics, Cincinnati Children’s Hospital Medical Center, 3333 Burnet Avenue, Cincinnati, OH 45229, United States; Department of Pediatrics, University of Cincinnati College of Medicine, 3230 Eden Avenue, Cincinnati, OH 45267, United States; Division of Pulmonary Medicine, Department of Pediatrics, Cincinnati Children’s Hospital Medical Center, Cincinnati, 3333 Burnet Avenue, OH 45229, United States; Division of Immunobiology, Department of Pediatrics, Cincinnati Children’s Hospital Medical Center, 3333 Burnet Avenue, Cincinnati, OH 45229, United States; Department of Molecular and Cellular Biology, Scripps Research, 10550 North Torrey Pines Road, La Jolla, CA 92037, United States

**Keywords:** Neurodevelopmental Disorder, DLG4, PSD-95, SHINE Syndrome, DLG4-Related Synaptopathy

## Abstract

SHINE syndrome is a rare neurodevelopmental disorder caused by mutations in *DLG4*, which encodes the postsynaptic scaffolding protein PSD-95. Key symptoms include sleep problems, hypotonia, intellectual disability, neurological disorders, and epilepsy, hence the name ‘SHINE.’ Here, we developed and characterized a mouse model of SHINE syndrome carrying the patient-derived DLG4^V692Wfs*12/+^ variant associated with a severe form of the disorder. The mutant transcript escapes nonsense-mediated decay but results in reduced PSD-95 protein expression, faithfully reproducing the molecular phenotype observed in the patient. Behavioral analyses revealed that Dlg4^V692Wfs*12/+^ mice recapitulate several hallmark features of SHINE syndrome, often in a sex-specific manner. Male mutants showed deficits in learning and cognitive flexibility. Dlg4^V692Wfs*12/+^ mice also demonstrate trends toward altered sensory processing and socialization. Male mutants exhibited an increased proportion of short sleep bouts and compensatory longer average sleep bout length, suggesting sporadic sleep reminiscent of the patient. While spontaneous seizures were not observed, future studies will test susceptibility to provoked seizures. Together, these findings establish Dlg4^V692Wfs*12/+^ mice as a robust and translationally relevant model that reproduces key molecular and behavioral features of SHINE syndrome. This model provides a valuable resource for elucidating the mechanisms underlying synaptic neurodevelopmental disorders and for identifying potential therapeutic strategies.

## Introduction

Sleep problems are pervasive in neurodevelopmental disorders (NDDs), but their importance, and contribution to morbidity, is often overlooked [1]. Sleep disorders can arise from disruptions in the circadian clock or sleep genes. The circadian clock, a 24-hour transcriptional-translational feedback loop in virtually all cells, aligns physiology with important external cues, such as daylight. External signals are integrated in the suprachiasmatic nucleus (SCN) of the hypothalamus, which then synchronizes clocks in other organs [2]. The hypothalamus also contains nuclei involved in sleep drive and arousal, such as in the ventrolateral preoptic nucleus and the lateral hypothalamus, respectively, making it a key sleep organ [3]. Improving sleep in patients with NDDs, either by treating the underlying cause or by administering hypnotics, can reduce severity of symptoms and improve quality of life for the patient and caregivers [1]. Here, we developed a novel mouse model to study sleep problems in SHINE syndrome based on a mutation identified in a patient with severe symptoms.

SHINE syndrome, also known as *DLG4*-related synaptopathy, is a rare NDD resulting from an autosomal-dominant mutation in the gene *discs large MAGUK protein 4* (*DLG4*) [4]. Key symptoms include sleep problems, hypotonia, intellectual disabilities, neurological disorders, and epilepsy, resulting in the name ‘SHINE’ [5]. Variants in *DLG4* were first reported in 2018 to cause a ‘Marfanoid-like intellectual disability’ [6], and were later identified in 2021 as an independent monogenic syndrome [4]. Over the past few years, several studies have been published describing diagnostic criteria and clinical management [5], characterizing clinical epilepsy phenotypes [7], and reporting a case study suggesting the association of a *DLG4* variant with treatment-resistant schizophrenia [8]. To date, ~ 235 individuals have been identified with SHINE syndrome worldwide [9]. These individuals show considerable variability in symptom severity, even among those with the same mutation [4]. There remains a large gap in our understanding of how *DLG4* variants produce the symptoms observed in SHINE syndrome.

The *DLG4* gene encodes postsynaptic density protein 95 (PSD-95), a scaffolding protein highly expressed on the postsynaptic membrane of excitatory neurons [10]. PSD-95 interacts with and promotes the function of many key receptors in the postsynaptic membrane, including directly binding to the NMDA and TRK-B receptors and indirectly binding AMPA receptors [11–14]. These functions make PSD-95 a prime target for the regulation of sleep at both molecular and circuit levels. Haploinsufficiency of PSD-95, as is observed in patients with SHINE syndrome, may result in impaired synaptic function and cause the many neurological symptoms observed in patients. However, there are currently no models for studying the molecular, physiological, and behavioral manifestations of SHINE syndrome, or for testing potential therapies.

Here, we generate a novel mouse model bearing the *Dlg4* p.(V692Wfs*12) mutation (Dlg4^V692Wfs*12/+^) and show that this model recapitulates molecular and behavioral phenotypes observed in a patient with the same mutation, including sleep problems. We demonstrate that the p.(V692Wfs*12) RNA in SHINE mice escapes nonsense mediated decay and that PSD-95 protein expression is decreased, similar to what we observe in our patient. While patients experience sleep, intellectual, and neurological dysfunction, SHINE mice exhibit sleep abnormalities, impaired learning and memory, and abnormal startle reflexes. These novel transgenic mice provide an excellent model and tool for researchers studying this rare NDD.

## Results

### DLG4 modulates circadian rhythms in vitro

The *DLG4* gene is both regulated by and capable of modulating the circadian clock. Circadian regulation can be evaluated at multiple levels, including molecular and locomotor activity rhythms. Molecular rhythms reflect the cyclic expression of core circadian clock genes, such as *BMAL1* and are present in nearly all vertebrate cells. By contrast, activity rhythms are assessed through long-term recordings of locomotor behavior. Publicly available ChIP-seq data indicate the binding of bHLH transcription factors, ARNT (NCBI GEO accession no. GSE127382) and BHLHE40 (NCBI GEO accession no. GSE106000), at E-box motifs in the *DLG4* promoter and structural gene (Fig. 1A and Fig. S1A). These motifs are binding sites for core circadian transcription factors (*CLOCK/BMAL1* at E-boxes and *ROR/REV-ERB* at ROREs), and their presence suggests that *DLG4* expression is under circadian control (Fig. S1B). Supporting this, a previously published gene expression microarray dataset from our lab (NCBI GEO accession no. GSE54652) revealed that *Dlg4* expression in the mouse SCN exhibits 24-hour rhythmicity, with expression peaking during the activity phase and decreasing during the resting phase (analyzed using JTK_CYCLE, *P* < .001, Fig. 1B) [15]. This rhythmic pattern is consistent with regulation by circadian–driven transcriptional mechanisms. In our published siRNA screen, we used the synchronized U2 OS cell model expressing a BMAL1::luciferase reporter to record real-time bioluminescence rhythms [16]. Knockdown of *DLG4* in this system shortened the circadian period *in vitro* (*P* < .001; Fig. 1C and D), indicating that *DLG4* contributes to circadian timekeeping. There was no difference in period between the sham siRNA treated cells and GFP+ cells (*P* = .77; Fig. 1D) [16, 17]. Beyond its well-established role in excitatory synapse communication, these findings raise the possibility that *DLG4* may, in part, influence sleep through its actions on the clock.

**Figure 1:**
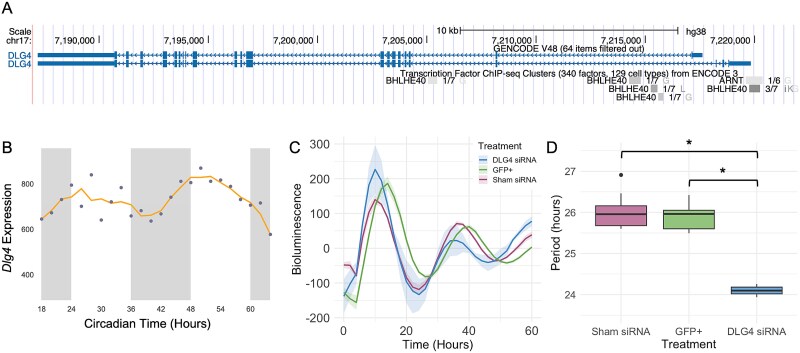
DLG4 *is implicated in circadian processes*. (A) ChIP-seq tracks from the UCSC genome browser show binding of the bHLH transcription factors ARNT and BHLHE40 across the human *DLG4* promoter, indicating the presence of functional E-box motifs. (B) Microarray analysis demonstrates rhythmic *Dlg4* expression in mouse SCN tissue (three mice sampled every 2 hours for 48 hours; rhythmicity determined using JTK_CYCLE, *P* < .001). (C) Representative bioluminescence traces from synchronized U2 OS cells expressing either GFP (green) or the BMAL1::Luciferase reporter treated with sham siRNA (pink) or *DLG4* siRNA (blue). Solid lines represent the mean; shaded areas indicate ± SEM. (D) Quantification of circadian period analyzed using the MetaCycle package in RStudio (sham siRNA, *n* = 6, 26.0 ± 0.13 hr; GFP+, *n* = 6, 25.9 ± 0.13 hr; *DLG4* siRNA, *n* = 2, 24.1 ± 0.27 hr; *P* < .001).

### A novel Dlg4^V692Wfs*12/+^ mouse recapitulates PSD-95 haploinsufficiency observed in the originating patient

We generated an avatar mouse model bearing the same p.(V692Wfs*12) variant found in a patient with severe SHINE syndrome. This patient exhibits all the hallmark symptoms of SHINE syndrome: sleep problems (difficulty falling asleep, frequent awakenings, abnormal sleep architecture on EEG, decreased REM sleep), hypotonia, severe intellectual disability, neurological problems (ataxia, stereotypies, abnormal sensory processing, comorbid autism spectrum disorder), and epilepsy (multifocal epileptiform discharges and electrical status epilepticus in sleep) (Table 1). The mutation is predicted to result in a truncated protein missing the latter portion of the guanylate kinase-like domain and C-terminus (Fig. 2A), possibly impairing the ability of PSD-95 to bind other components of the post-synaptic density [18]. CRISPR editing was used to develop mice heterozygous for the NM_007864.3:c.2074_2078delGTAGAinsT variant (Fig. 2B). Previous publications demonstrated that this DLG4^V692Wfs*12/+^ variant (reported as p.(V735Wfs*12) using the minor PSD-95β isoform) escapes nonsense mediated decay and results in PSD-95 haploinsufficiency in the patient [4, 7]. Using allele-specific probes for wild-type and mutant *Dlg4* cDNA, we found that the variant transcript is expressed in the cerebellum (*P* = .0016; Fig. 2C) and hypothalamus (Fig. S2A) of Dlg4^V692Wfs*12/+^ mice, thereby escaping nonsense mediated decay. Protein quantification of cerebellar synaptosomes using mass spectrometry showed a reduction in PSD-95 protein levels in Dlg4^V692Wfs*12/+^ mice compared to wild-type littermates (*P* = .047; Fig. 2D). This reduction is also seen on Western blot (Fig. S2B). These findings show that the novel mouse model mirrors the molecular and biochemical phenotype observed in the patient carrying the same variant [4, 7].

**Table 1:** Patient profile.

ID	CIRC_0249
Sex	male
Age of Onset	18 months
**Variant**	
ClinVar Variation ID	1 329 907
Inheritance	*de novo*
gDNA Chr17 (GRCh38)	g.7190805_7190809delGTGGAinsA
cDNA NM_001321075.3	c.2074_2078delGTGGAinsT
Coding Effect	frameshift
Exon	20
Domain	guanylate kinase-like domain
Protein Effect	p.(Val692Trpfs*12)
Classification	pathogenic
**Phenotype**	
Developmental Delay	+
Developmental Regression	2–5 years
Intellectual Disability	severe
Epilepsy	electrical status epilepticus in sleep multifocal epileptiform discharges
Sleep	difficulty falling asleep frequent awakenings abnormal sleep architecture on EEG low (11.5%) REM sleep
Sensory Processing	low registration: high neurological thresholds, passive self-regulation strategy
Autism Spectrum Disorder	+
Hypotonia	+
Stereotypies	+
Ataxia	+
MRI	normal

**Figure 2:**
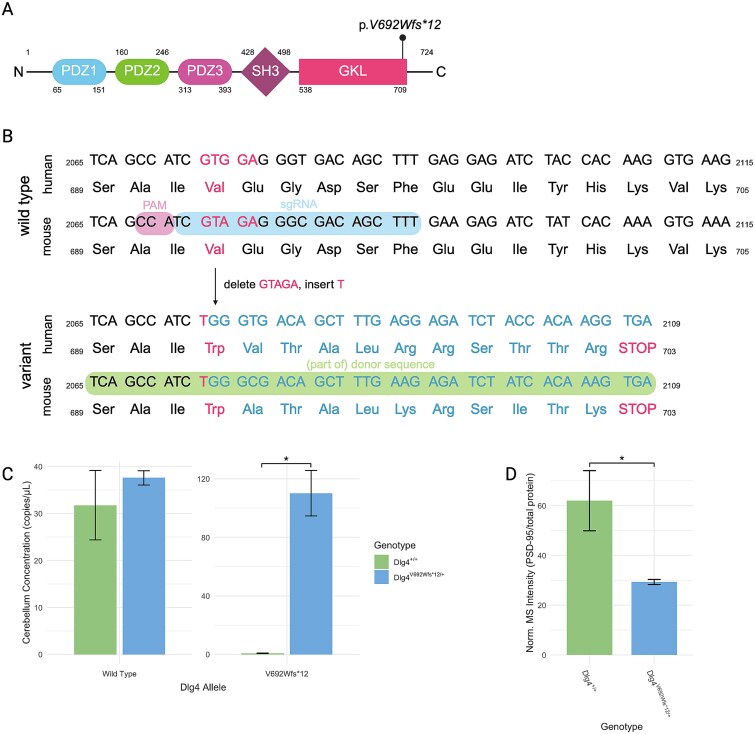
*Development of a novel* Dlg4^V692Wfs*12/+^  *mouse model*. (A) Schematic of the PSD-95 protein encoded by *DLG4*, indicating the location of the p.(V692Wfs*12) frameshift variant within the C-terminal guanylate kinase-like (GKL) domain. (B) Alignment of wild-type and variant coding sequences, with the CRISPR/Cas9 editing strategy shown (PAM sequence, pink; sgRNA, blue; donor sequence, green). (C) Gene expression of wild-type and p.(V692Wfs*12) *Dlg4* alleles in the cerebellum of *Dlg4^+/+^* and Dlg4^V692Wf*s12/+^ mice using digital PCR (V692Wfs*12; *Dlg4^+/+^*, 0.795 ± 0.19 copies/μL; Dlg4^V692Wfs*12/+^, 110.14 ± 15.52 copies/μL; *P* = .0016). (D) PSD-95 mass spectrometry intensity normalized to total protein intensity in cerebellar synaptosomes of *Dlg4^+/+^* and Dlg4^V692Wf*s12/+^ mice (*Dlg4^+/+^*, 61.96 ± 12.09; Dlg4^V692Wfs*12/+^, 29.30 ± 1.01; *P* = .047). Data represent mean ± SEM from *Dlg4^+/+^* (*n* = 3 males) and Dlg4^V692Wfs*12/+^ (*n* = 3 males).

### A novel Dlg4^V692Wfs*12/+^ mouse model shows cognitive and neurological behavioral symptoms observed in the originating patient

Behavioral characterization of the Dlg4^V692Wfs*12/+^ mice revealed that they recapitulate several key patient symptoms, often in a sex-specific manner, with males showing a more severe phenotype. Because epilepsy is a major symptom in SHINE syndrome, we were interested in assessing for spontaneous seizures in the Dlg4^V692Wfs*12/+^ mice. Mice were anesthetized and implanted with bilateral cortical electrodes and a ground electrode connected to a wireless transmitter. Continuous video-EEG monitoring was recorded for two weeks. No spontaneous seizures were observed in the Dlg4^V692Wfs*12/+^ nor the *Dlg4^+/+^* mice. This discrepancy may arise from differences in genetic background between humans and mice, as well as the relatively low-stimulus environment in which the mice are housed. Further considerations and future directions are discussed in the Discussion section.

Analogous to assessing intellectual disability in patients, the Morris Water Maze assay is a classical paradigm for measuring learning and memory deficits in mice. Dlg4^V692Wfs*12/+^ mice exhibited longer escape latencies than controls during training trials (*P* = .0049; Fig. 3A), as well as impaired path efficiency on day 5 of acquisition and days 3 and 5 of reversal (Fig. S3A), indicating impaired learning. There was no overall difference in swimming distance (*P* = .58) or speed (*P* = .20), suggesting that any changes in performance are not due to motor impairment (Fig. S3B and C). Probe trials of the Morris Water Maze assay revealed that during reversal, male Dlg4^V692Wfs*12/+^ mice take longer to enter the target quadrant compared to controls (*P* = .025; Fig. 3B). Additionally, during reversal, male Dlg4^V692Wfs*12/+^ mice entered the target quadrant significantly fewer times than controls (*P* = .0078), and, interestingly, female mice entered significantly more times than controls (*P* < .001; Fig. S3D). There was no difference in distance traveled during probe trials (*P* = .73; Fig. S3E). Together, these data demonstrate distinct impairments in learning and cognitive flexibility in male Dlg4^V692Wfs*12/+^ mice, recapitulating the intellectual disability observed in the male patient.

**Figure 3:**
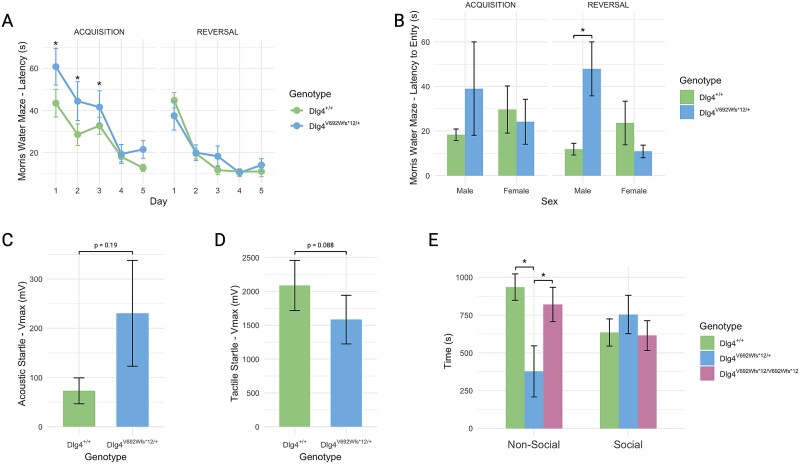
*Behavioral phenotypes of a novel mouse model mirror patient symptoms*. (A) Latency to reach the platform during Morris water maze learning trials (genotype effect, *P* = .005; day 1, *P* < .001; day 2, *P* < .001; day 3, *P* = .007). (B) Latency to enter the target quadrant during Morris water maze probe trials (reversal; *Dlg4^+/+^* males, 47.9 ± 12.1 s; Dlg4^V692Wfs*12/+^ males, 11.9 ± 2.6 s; *P* = .025). (C) Acoustic startle response (V_max_; *Dlg4^+/+^*, 73 ± 97 mV; Dlg4^V692Wfs*12/+^, 230 ± 143 mV; *P* = .19). (D) Tactile startle response (V_max_; *Dlg4^+/+^*, 2087 ± 276 mV; Dlg4^V692Wfs*12/+^, 1583 ± 319 mV; *P* = .088). Data represent mean ± SEM from *Dlg4^+/+^* (*n* = 10; 5 males, 5 females) and Dlg4^V692Wfs*12/+^ (*n* = 8; 3 males, 5 females) mice. (E) Time spent in non-social and social chambers during the social choice assay (*Dlg4^+/+^*, 935 ± 118 s; Dlg4^V692Wfs*12/+^, 377 ± 118 s, *P* < .05; Dlg4^V692Wfs*12/V692Wfs*12^, 820 ± 118 s). Data represent mean ± SEM from *Dlg4^+/+^* (*n* = 6; 3 males, 3 females), Dlg4^V692Wfs*12/+^ (*n* = 6; 2 males, 4 females), and Dlg4^V692Wfs*12/V692Wfs*12^ (*n* = 6, 3 males, 3 females).

In order to assess for neurological abnormalities in Dlg4^V692Wfs*12/+^ mice, we assayed for acoustic and tactile startle reflex, as well as three-chamber social choice and accelerating rotarod. The V_max_ response to acoustic startle was trending upwards in Dlg4^V692Wfs*12/+^ mice compared to controls (*P* = .19; Fig. 3C), suggesting heightened responses to central stimuli. On the other hand, there was a trending decrease in tactile reflex response in Dlg4^V692Wfs*12/+^ mice (*P* = .088; Fig. 3D), suggesting impaired responses to peripheral stimuli. There were no observed differences in prepulse inhibition between the two groups (59–93 dB; all *P* > .15; Fig. S4A). These trends parallel observations in the patient: impaired sensory processing with low registration may correspond to reduced tactile startle responses, while a tendency toward central hyperexcitation, manifesting clinically as epilepsy, may align with enhanced acoustic startle responses (Table 1).

Three-chamber social choice testing was used to assess autism spectrum disorder (ASD)-like behavior in the mice. Heterozygous mice exhibited a decreased preference for the non-social chamber (*P* < .05), while wild-type and homozygous Dlg4^V692Wfs*12/V692Wfs*12^ mice demonstrated similar social preferences (*P* = 1.00; Fig. 3E). Genotype significantly affected the amount of time mice spent in the chambers (*P* = .018), with a trend toward a genotype × chamber interaction (*P* = .062), suggesting that some genotypes may show chamber-specific preferences or avoidance (Fig. S4B). Although the overall difference in preference index for the social chamber was not statistically significant (*P* = .064), there was a trend toward increased social preference in heterozygous mice compared to both wild-type and homozygous mice (*P* > .1; Fig. S4C). This observation is consistent with prior work in *Dlg4^+/−^* mice, which also exhibited hypersocialization in the free social interaction test (Table 2) [19]. In contrast, studies of *Dlg4^−/−^* mice have reported conflicting results, with some describing hypersocialization and others hyposocialization (Table 2) [20, 21]. Taken together, these findings suggest that *Dlg4* mutations lead to abnormal socialization in mice, manifested either as excessive, inappropriate interaction (hypersocialization) or social withdrawal (hyposocialization), both of which reflect autistic-like social deficits. This aligns with recent evidence identifying *Dlg4* downregulation as an autism risk factor in children [23].

**Table 2:** Comparison of behavioral assays in published Dlg4 transgenic mice.

Mouse Model	*Dlg4* ^V692Wfs*12/+^	*Dlg4* ^*+/−*^	*Dlg4* ^*−/−*^
**Learning & Memory**			
Morris Water Maze	*Impaired learning*: ↑ escape latency, ↓ path efficiency *Impaired cognitive flexibility (males):* ↑ latency to entry, ↓ entries to target quadrant	Normal [19]	No data
Novel Object Recognition	No data	No data	Normal [20]
Discrete-Trial T-Maze	No data	No data	↓ spontaneous alternation [20, 21] Deficit in delayed tasks [20]
Associative Learning Tasks	No data	No data	Impaired operant, pavlovian conditioning [22]
**Startle Reflex**			
Tactile Startle	Normal (trending ↓)	No data	No data
Acoustic Startle	Normal (trending ↑)	No data	Normal (trending ↓) [21]
**Sociability**			
Social Choice	Normal (trending hypersocialization)	No data	Hypersocialization [21] Hyposocialization [20]
Free Social Interaction		Hypersocialization [19]	Normal [21]
Novel Mouse	No data	No data	↓ preference for novel mouse [20]
Vocalization	No data	↓ latency to vocalization in females, ↑ vocalization in females [19]	↑ latency to vocalization in males, ↓ vocalizations in males [21]
**Motor Coordination**			
Accelerating Rotarod	Normal	Normal [19]	↓ latency [21]
Balance Beam	No data	Normal [19]	↑ foot-slips on narrowest beam [21]
Inverted Wire-Hang	No data	No data	↓ latency [21]
**Locomotor Activity**			
Wheel Running	Normal	No data	No data
Open Field Test	No data	Hypoactivity [19]	Hypoactivity [21] Normal [20]
Home Cage Observation	No data	Normal [19]	Normal [21]

Several patients with SHINE syndrome, including the individual carrying the p.(V692Wfs*12) variant, experience developmental regression leading to loss of ambulation [5]. To assess motor impairments in our mouse model, we used the accelerating rotarod. Accelerating rotarod tests revealed an unexpected age-related phenotype. 2-month old Dlg4^V692Wfs*12/+^ mice exhibited decreased latency on day 1 (*P* = .029), but this difference vanished on day 2 of testing (*P* = .91; Fig. S4D). Additional testing of 4 month-old (*P* = .59) and 6 month-old mice (*P* = .33) revealed no significant genotype differences in accelerating rotarod performance (Fig. S4E and F). These data are consistent with the Morris Water Maze results that found no motor impairments (Fig. S3C). These results are consistent with prior studies of *Dlg4^+/−^* mice, but differ from *Dlg4^−/−^* mice, which exhibit persistent motor impairments across multiple assays, including the accelerating rotarod, balance beam, and inverted wire-hang tests (Table 2) [19, 21].

All together, these behavioral assays demonstrate that male Dlg4^V692Wfs*12/+^ mice exhibit memory and learning impairments and may also recapitulate some sensory reflex and social phenotypes observed in the patient.

### A novel Dlg4^V692Wfs*12/+^ mouse recapitulates locomotor and sleep behaviors observed in the originating patient

Many SHINE patients experience sleep problems, so we were interested in evaluating circadian and sleep parameters in our novel Dlg4^V692Wfs*12/+^ mice. To accomplish this, we tested 15 wild-type (7 male, 8 female) and 17 Dlg4^V692Wfs*12/+^ (9 male, 8 female) mice, aged 7–11 weeks, using wheel-running and PiezoSleep assays following the protocol shown in Fig. 4A. For both assays, the mice were given at least 7 days to acclimate to the new environment and then were recorded in a 12:12 hour light–dark (LD) cycle for at least 7 days. Light conditions were then switched to constant darkness (DD) for 14 days and returned to 12:12 hour light–dark (LD2) for another 7 days. In the PiezoSleep assay, mice were additionally exposed to a 6-hour phase advance for 14 days and subsequently to a 6-hour phase delay for 14 days. Due to space constraints, not all mice could be tested using wheel running or PiezoSleep at the same time; therefore, female mice started on wheel running and male mice started on PiezoSleep and then were switched once the testing protocol was completed.

**Figure 4:**
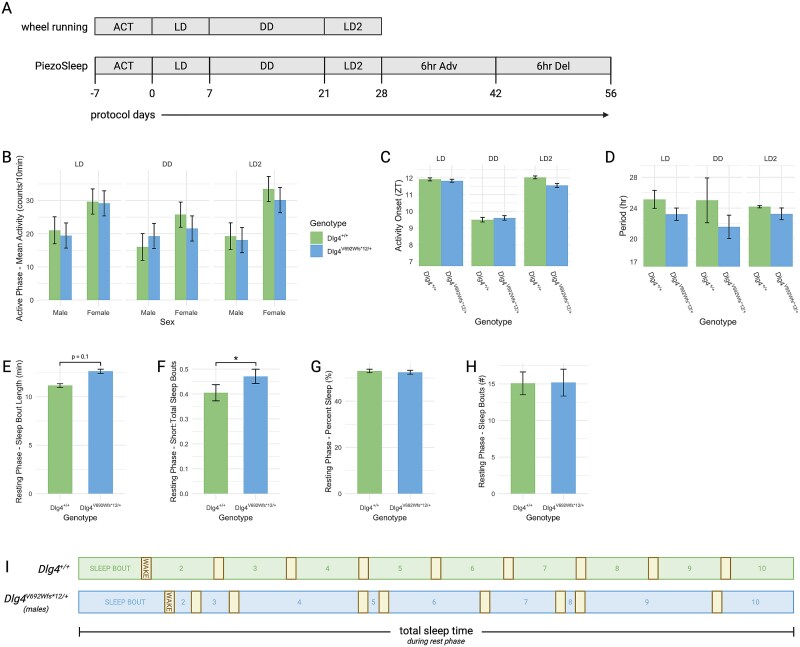
Activity and sleep changes in the *Dlg4*^V692Wfs*12/+^ mouse model. (A) Protocol for wheel running and PiezoSleep assays (ACT, acclimatize; LD, 12:12 hour light–dark; DD, constant darkness, LD2, 12:12 hour light–dark; 6 hr Adv, 6-hour phase advance; 6 hr Del, 6-hour phase delay). (B) Mean activity counts during wheel running in the active phase (*P* = .62). (C) Activity onset during wheel running (*P* = .29). (D) Period length of locomotor activity during wheel running (*P* = .09). (E) Mean sleep bout length during PiezoSleep recording in the resting phase (*Dlg4^+/+^*, 11.2 ± 0.58 min; Dlg4^V692Wfs*12/+^, 12.6 ± 0.59 min; *P* = .10). (F) Ratio of short (≤ 2 min) to total number of sleep bouts during PiezoSleep recording in the resting phase (*Dlg4^+/+^* males, 0.503 ± 0.02; Dlg4^V692Wfs*12/+^ males, 0.435 ± 0.02; *P* = .035). (G) Percent of time spent asleep during PiezoSleep recordings in resting phase (male mice, *P* = .80). (H) Total number of sleep bouts during PiezoSleep recordings in resting phase (male mice, *P* = .97). (I) Schematic depicting sleep changes observed in male Dlg4^V692Wfs*12/+^ mice compared to controls. Data represent mean ± SEM from *Dlg4^+/+^* (*n* = 15; 7 males, 8 females), Dlg4^V692Wfs*12/+^ (*n* = 17; 9 males, 8 females).

Wheel running is a well-established assay for quantifying locomotor activity and circadian rhythms in rodents. As a voluntary and motivated behavior, it provides a robust measure of overall activity as well as the active and rest phases. Wheel running revealed normal activity in Dlg4^V692Wfs*12/+^ mice (*P* = .62; Fig. 4B). However, traces of wheel running activity demonstrated a trend towards mild global hypoactivity in Dlg4^V692Wfs*12/+^ mice except for males in constant darkness (Fig. S5A). Interestingly, Dlg4^V692Wfs*12/+^ male mice were particularly hyperactive right before their resting phase, recapitulating hyperactivity demonstrated by the patient before his sleep phase as observed using actigraphy (*yellow boxes in*  Fig. S5A and B). There was no difference in activity onset (*P* = .2886; Fig. 4C) or period (*P* = .086; Fig. 4D). Actigraphy data from the patient also demonstrate a normal period (1440 minutes [24 hours], *P* < .05; Fig. S5C). Overall, the wheel running data show similar activity patterns between the patient and Dlg4^V692Wfs*12/+^ male mice.

In order to assess sleep non-invasively in our mouse model, we used PiezoSleep monitoring sensors. These sensors capture gross body movements and respiratory patterns, enabling automated classification of sleep and wake states [24, 25]. Dlg4^V692Wfs*12/+^ mice exhibited a trend toward longer average sleep bouts during the resting phase relative to controls (*P* = .101; Fig. 4E). Simultaneously, there is an increased ratio of short (≤ 2 min) to total sleep bouts in Dlg4^V692Wfs*12/+^ male mice (*P* = .035; Fig. 4F, Fig. S6A). The Dlg4^V692Wfs*12/+^ male mice exhibited no differences in the percent of resting phase spent asleep (*P* = .802; Fig. 4G) or in the total number of sleep bouts during the resting phase (*P* = .971; Fig. 4H). This suggests that Dlg4^V692Wfs*12/+^ male mice have normal total sleep duration and number of sleep bouts, but that many of those sleep bouts are short and are interspersed by compensatory longer sleep bouts (Fig. 4I). This increased proportion of short sleep bouts could result in decreased time spent in REM sleep, which would align with low REM sleep observed in the patient on sleep studies (Table 1) [26]. A phase shift protocol revealed no differences in realignment between Dlg4^V692Wfs*12/+^ and *Dlg4^+/+^* mice (*P* = .854; Fig. S6B and C). In sum, these data show that the Dlg4^V692Wfs*12/+^ variant in male mice recapitulates locomotor and sporadic sleep phenotypes observed in the patient.

## Discussion

We developed a model of SHINE syndrome, carrying the patient-derived Dlg4^V692Wfs*12/+^ variant associated with a severe form of SHINE. This mutation was sufficient to reproduce several key molecular and behavioral features of SHINE syndrome. Dlg4^V692Wfs*12/+^ mice recapitulated the patient’s molecular phenotype: mutant transcripts escaped nonsense-mediated decay, and PSD-95 protein expression was reduced (Table 1; Fig. 2C and D, Fig. S2A and B) [4, 7]. Male Dlg4^V692Wfs*12/+^ mice exhibited deficits in learning, memory, and sleep that parallel the intellectual disability and sleep disturbances observed in patients (Table 1; Fig. 3A and B; Fig. S3; Fig. 4, Fig. S6A). They also showed a trend toward hypersocialization, indicative of autism-like behavior, and abnormal sensory reflexes, consistent with impaired sensory processing reported in patients (Table 1; Fig. 3C and D; Fig. S4A). Because animal models are critical for dissecting the mechanisms underlying neurodevelopmental symptoms, this model offers a valuable resource for investigating SHINE syndrome.

Our avatar mouse model deviates from previously characterized *Dlg4^+/−^* and *Dlg4^−/−^* models in several key ways, indicating that the patient-derived allele produces effects distinct from gene deletion. Notably, Dlg4^V692Wfs*12/+^ mice exhibited impairments in learning and memory, whereas *Dlg4^+/−^* mice showed no such abnormalities (Fig. 3A and B; Table 2) [19]. This suggests that the patient allele may exert a more severe functional disruption than gene deletion, contributing to the intellectual disability observed in SHINE syndrome. Moreover, Dlg4^V692Wfs*12/+^ mice demonstrated a trend toward increased acoustic startle reactivity, indicative of central sensitization, while *Dlg4^−/−^* mice instead displayed a trend toward desensitization (Table 2) [21]. Together, these findings support the conclusion that the patient-derived variant does not simply recapitulate *Dlg4* loss, but rather produces molecular and behavioral phenotypes reflecting human disease.

The avatar mouse model aligns with previously reported *Dlg4^+/−^* phenotypes in sociability and motor coordination. Dlg4^V692Wfs*12/+^ mice showed no significant differences in social behavior but exhibited a clear trend toward hypersocialization (Fig. 3E; Fig. S4B and C), consistent with prior findings in *Dlg4^+/−^* mice (Table 2) [19]. In contrast, studies of *Dlg4^−/−^* mice have reported mixed outcomes, ranging from hyper- to hypo-social behavior (Table 2) [20, 21]. Together, these results suggest that loss or disruption of *Dlg4* perturbs normal social behavior, resulting in either excessive or reduced social interaction, both indicative of autism-related social deficits. This is consistent with recent evidence associating decreased *DLG4* expression with autism in children [23]. Unexpectedly, Dlg4^V692Wfs*12/+^ mice exhibited normal motor coordination, paralleling *Dlg4^+/−^* mice (Table 2), but differing from *Dlg4^−/−^* mice, which display impaired coordination relative to controls (Table 2) [20, 21]. These findings highlight that the patient-derived allele produces selective behavioral alterations, rather than the broad impairments observed in null and heterozygous loss-of-function models.

While mouse models are powerful for investigating the genetic basis of behavior, disease mechanisms, and therapeutic strategies, they cannot fully recapitulate the complexity of human conditions [27]. Although Dlg4^V692Wfs*12/+^ mice exhibited normal motor coordination consistent with *Dlg4^+/−^* models, this contrasts with the clinical presentation of SHINE syndrome. Many patients, including the individual carrying the p.(V692Wfs*12) variant, experience developmental regression resulting in motor impairments, sometimes progressing to loss of ambulation (Table 1) [5]. Furthermore, despite seizures being a prominent feature of SHINE syndrome [5], Dlg4^V692Wfs*12/+^ mice did not exhibit spontaneous seizures during two weeks of continuous monitoring. This may reflect the relatively low-stimulus housing environment compared with human conditions [27]. Future studies should test whether environmental stressors or proconvulsant agents such as kainic acid can elicit seizures, which would clarify whether Dlg4^V692Wfs*12/+^ mice exhibit increased neuronal excitability or a reduced seizure threshold. Identifying seizure triggers in patients will further refine the translational relevance of this model. Overall, these findings underscore the value of patient avatar models, which complement null mutants by capturing variant-specific disease mechanisms critical for understanding rare neurodevelopmental disorders like SHINE syndrome.

In conclusion, our findings suggest that the patient-derived p.(V692Wfs*12) allele does not behave as a simple loss-of-function variant but rather exhibits features consistent with a dominant-negative mechanism. Despite reduced PSD-95 protein levels, Dlg4^V692Wfs*12/+^ mice exhibit more severe molecular and behavioral phenotypes than *Dlg4^+/−^* mice. This implies that, even if expressed at low levels, the variant protein may interfere with normal synaptic function or PSD complex formation. Future studies of this mouse model should include evaluation of gene and protein expression of the variant allele, global synaptic protein expression, and synaptic function. These results also highlight the need to model additional patient-derived variants to better define the mechanisms and phenotypic variability of SHINE syndrome. Together, these findings indicate that SHINE syndrome pathogenesis may involve complex molecular interactions beyond simple haploinsufficiency, underscoring the importance of patient-derived alleles for uncovering the nuanced mechanisms underlying this disorder.

## Materials and methods

### Ethics statement

Human data were obtained through the Sleep and Circadian Disorders Registry at CCHMC (IRB #2020–0402), with written informed consent provided by the participant’s parents. All animal studies and experimental protocols were approved by the Institutional Animal Care and Use Committee (IACUC) at the authors’ institution (protocol #2025–0037) and were conducted in accordance with applicable national and institutional guidelines for the care and use of laboratory animals.

### Generation of patient avatar of SHINE

A novel mouse model was generated in collaboration with the Transgenic Animal and Genome Editing (TAGE) Facility at CCHMC. Using CRISPR/Cas9-mediated genome editing, a targeted mutation was introduced into the mouse genome to model the human SHINE syndrome-associated variant. Guide RNAs and donor templates were designed and validated by the TAGE Facility, and zygote microinjection was performed to generate founder animals. Positive founders were identified via PCR genotyping and confirmed by Sanger sequencing. The resulting mice were backcrossed to a C57BL/6 J background for at least two generations before experimental use.

### RNA isolation, digital PCR, and allele-specific PCR

Total RNA was extracted from cerebellum and hypothalamus tissue of SHINE and wild-type mice using standard protocols. RNA was reverse-transcribed into complementary DNA (cDNA) using the High-Capacity cDNA Reverse Transcription Kit (*Applied Biosystems, 4 368 814*) according to the manufacturer’s instructions. Digital PCR was performed using the QuantStudio Absolute Q Digital PCR System (*Applied Biosystems, A52864*) in combination with the mouse TaqMan SNP Genotyping Assay (*Applied Biosystems, 4 351 384*) targeting the wild-type and p.(V692Wfs*12) *Dlg4* alleles. Reactions were prepared using cDNA and Absolute Q Universal DNA Digital PCR Master Mix (*Applied Biosystems, A72710*) according to the manufacturer’s instructions and loaded onto microfluidic array plates (QuantStudio Absolute Q MAP16 Plate; *Applied Biosystems, A52865*) for partitioning and amplification. Following thermal cycling, endpoint fluorescence was measured and analyzed using the QuantStudio Absolute Q Digital PCR Software to quantify allele-specific signal based on probe fluorescence. Data were expressed as copies/μL. Allele-specific PCR was performed using primers targeting the wild-type and V692Wfs*12 DLG4 alleles. Amplification was conducted with EconoTaq Green DNA Polymerase (*Lucigen*) for 25 cycles under standard thermocycling conditions. PCR products were separated on a 1% agarose gel containing SYBR Safe DNA Gel Stain (*Thermo Fisher Scientific, S33102*) and visualized under UV illumination.

### Mass spectrometry

Synaptosomes were isolated from cerebellar tissue of *Dlg4*^+/+^ and Dlg4^V692Wfs*12/+^ mice using SynPER Synaptic Protein Extraction Reagent (*Thermo Scientific, 87 793*) according to the manufacturer’s protocol, and protein concentration was determined using a BCA assay. Mass spectrometry was completed in the Prasad lab following established workflows. Samples were subjected to in-solution tryptic digestion followed by peptide cleanup using standard protocols. Peptides were analyzed by liquid chromatography–tandem mass spectrometry, with data acquired in data-dependent mode and singly charged ions excluded from fragmentation. Raw data were processed using MaxQuant (*version 1.6.8.0; Max Planck Institute of Biogeochemistry, Germany*) and matched against the UniProt *Mus musculus* reference proteome via the Andromeda search engine. A decoy database approach was used to control false discovery rates, with peptide and protein FDR set at 1%, and up to two missed tryptic cleavages allowed.

### Western blot analyses

Protein was extracted from dissected cerebellum tissue using RIPA buffer supplemented with protease and phosphatase inhibitors. Lysates were quantified using a BCA assay, and equal amounts of protein (10 μg) were separated by SDS-PAGE and transferred onto PVDF membranes. Membranes were blocked with 5% bovine serum albumin in TBST for 1 hour at room temperature, then incubated overnight at 4°C with primary antibodies: anti-PSD-95 (*1:2000; Millipore, MABN68*) and anti-GAPDH (*1:5000; Abcam, ab8245*). After washing, membranes were incubated with HRP-conjugated secondary antibodies for 1 hour at room temperature and developed using the Clarity Western ECL Substrate (*Bio-Rad, #170–5061*). Band intensities were quantified using ImageJ and normalized to GAPDH.

### Rotarod testing

Motor coordination and balance were assessed using an accelerating rotarod apparatus in collaboration with the Animal Behavioral Facility at CCHMC. Mice were placed on a rotating rod that gradually increased in speed from 4 to 40 rpm over a 5-minute trial. The latency to fall was recorded over four trials per day across two consecutive days. Trials were averaged to assess motor performance and learning over time. For the 2-month-old (2mo) cohort, 10 wild-type (5 males, 5 females) and 8 *Dlg4^V692Wfs12/+^* mice (3 males, 5 females) were tested. The 4-month-old (4mo) cohort included 19 wild-type (9 males, 10 females) and 18 *Dlg4^V692Wfs12/+^* mice (8 males, 10 females). The 6-month-old (6mo) cohort included 7 wild-type unrelated (UR; 4 males, 3 females), 8 wild-type littermates (4 males, 4 females), 8 *Dlg4^V692Wfs12/+^* (4 males, 4 females), and 8 Dlg4^V692Wfs12/V692Wfs*12^ mice (4 males, 4 females).

### Acoustic and tactile startle testing

Startle reactivity was assessed using a startle response system that measures whole-body movement in response to sensory stimuli as previously described [28, 29]. 10 wild-type mice (5 males, 5 females) and 8 Dlg4^V692Wfs*12/+^ mice (3 males, 5 females) were assessed in the Animal Behavioral Facility at CCHMC. Mice were placed in a sound-attenuated chamber and acclimated before testing. Acoustic startle responses were elicited with a 120 dB auditory pulse, and additional lower-intensity pulses (59, 70, 80, and 93 dB) were used to assess prepulse intensity–dependent modulation. Tactile startle was elicited using 60 psi air puffs directed at the animal. The maximum startle response amplitude, V_max_, evoked by a startle stimulus, is measured as the peak voltage (mV) generated by the animal’s whole-body movement in response to the acoustic or tactile stimulus. Startle amplitude was recorded over 2 days for 10 trials per day to evaluate sensorimotor gating and reactivity.

### Morris water maze

Spatial learning and memory were assessed using the Morris Water Maze in the Animal Behavioral Facility at CCHMC as previously described [30]. During the acquisition phase, mice were trained to locate a hidden platform submerged in a circular pool of opaque water using distal visual cues placed around the testing room. We tested 10 wild-type mice (5 males, 5 females) and 7 Dlg4^V692Wfs*12/+^ mice (2 males, 5 females). Each mouse completed one trial per day across five consecutive days, and performance was measured by escape latency (latency to reach the platform), path efficiency, distance, and speed. Following acquisition, mice underwent a reversal phase in which the platform was relocated to the opposite quadrant, requiring suppression of the previously learned location and acquisition of a new spatial association. Probe trials without the platform were conducted after both acquisition and reversal training to assess memory retention, measured as the latency to enter the target quadrant, number of entries into the target quadrant, and distance.

### E‌EG electrode implantation and recording

EEG monitoring was conducted in collaboration with the Danzer Lab at CCHMC as previously reported [31–33]. Mice were anesthetized with 2% isoflurane and implanted with bilateral anterior and posterior epidural cortical screw electrodes (1.60 mm and − 1.0 mm bregma, 1.80 mm and 4.8 mm lateral, respectively) and a ground electrode (−10 mm bregma, 1.0 mm lateral). Electrodes were secured to the skull with dental cement (*Parkell*). Continuous video-EEG monitoring was performed for two weeks (*PowerLab, AD Instruments*), and recordings were analyzed with Neuroscore software. Electrographic seizures were defined as repetitive spike discharges evolving into high-amplitude bursts lasting ≥10 seconds. We recorded 6 wild-type (3 males, 3 females) and 6 Dlg4^V692Wfs*12/+^ mice (3 males, 3 females). No seizures were observed during the monitoring period.

### Social choice testing

Social behavior was assessed using a three-chamber social choice assay. We tested 6 wild-type mice (3 males, 3 females), 6 Dlg4^V692Wfs*12/+^ mice (2 males, 4 females), and 6 Dlg4^V692Wfs*12/V692Wfs*12^ mice (3 males, 3 females). Mice were placed in a rectangular arena divided into three chambers and allowed to habituate for 10 minutes. During the test phase, an unfamiliar age- and sex-matched wild-type (social stimulus) was placed in a cage in one side chamber, and an identical empty cage (object stimulus) was placed in the opposite chamber. The test mouse was allowed to freely explore all three chambers for 10 minutes. Time spent in each chamber was recorded by overhead video and analyzed to assess sociability.

### Wheel running assay

To assess circadian locomotor activity, mice were individually housed in light-controlled environmental cabinets equipped with running wheels (*Actimetrics, Wilmette, IL*), and activity was recorded as previously described [34]. Wheel rotations were continuously recorded in 1-minute bins using automated data acquisition software. Mice acclimatized for 7 days and then were recorded in a 12:12 hour light–dark (LD) cycle for at least 7 days. This was followed by release into constant darkness (DD) for 14 days to evaluate free-running behavior. Next, mice were returned to a 12:12 hour light–dark (LD2) cycle for at least 7 days. We tested 15 wild-type mice (7 males, 8 females) and 16 Dlg4^V692Wfs*12/+^ mice (8 males, 8 females). Activity data were analyzed using ClockLab (*Actimetrics, Wilmette, Illinois, USA*) and RStudio to determine period length [35], activity counts, and activity onset [36].

### Sleep assay

To assess sleep–wake behavior noninvasively, mice were individually housed in cages equipped with PiezoSleep monitoring sensors (*Signal Solutions LLC, Lexington, Kentucky, USA*) and recorded as previously described [37]. The piezoelectric sensors detect gross body movements and respiratory patterns, enabling automated classification of sleep and wake states [24, 25]. Data were continuously recorded in 2-second epochs and processed using SleepStats (*Signal Solutions LLC, Lexington, Kentucky, USA*) software with default filtering parameters. We tested 15 wild-type mice (7 males, 8 females) and 16 Dlg4^V692Wfs*12/+^ mice (8 males, 8 females). Mice followed the same LD, DD, and LD2 cycle described in the wheel running assay with the addition of a 6-hour phase advanced (6 hr Adv) protocol for 14 days, followed by a 6-hour phase delay (6 hr Del) for another 14 days. Sleep metrics, including total sleep time, sleep bout duration, and sleep fragmentation, were extracted for analysis across light–dark and circadian conditions.

### Patient Actigraphy

The patient was recruited to the Sleep and Circadian Disorders Registry at CCHMC (IRB 2020–0402). As part of the registry, the patient was administered an actigraphy watch (*ActTrust 2, Condor Instruments, São Paulo, Brazil*) to track motion and sleep for at least four weeks. The ActTrust 2 is a wrist-worn device that records movement (via tri-axial accelerometry), wrist temperature, and light exposure, allowing characterization of sleep–wake and circadian rhythms. Data were stored internally on the device and later downloaded for analysis using the manufacturer’s software and the *actverse* RStudio package [38].

### Statistical analyses

For behavioral datasets involving repeated measures across multiple conditions or timepoints, we used linear mixed-effects models to account for both fixed and random effects [39]. Fixed factors typically included genotype, sex, and experimental conditions (e.g. day, pulse level, or trial type), while subject was modeled as a random effect to account for within-subject dependencies across repeated trials. Models were estimated using restricted maximum likelihood, and statistical significance of fixed effects was assessed with Type III ANOVA using Kenward–Roger approximation for denominator degrees of freedom. When significant main effects or interactions were detected, post hoc pairwise comparisons of estimated marginal means were performed with adjustments for multiple testing (e.g. Tukey or Holm) [40].

For other behavioral paradigms where the data did not conform to normality assumptions, such as activity onset, data were averaged across days per subject and analyzed using non-parametric approaches such as aligned rank transform ANOVA [36]. In cases where within-subject comparisons were central (e.g. social vs. non-social preference tests), repeated-measures ANOVA was applied with chamber as the within-subject factor and genotype as the between-subject factor. Across analyses, results were reported as mean ± SEM, and correction methods (Tukey or Bonferroni) were consistently applied to control for inflated Type I error rates in multiple comparisons. Oscillations in microarray gene expression datasets previously published by our lab were analyzed using Jonckheere–Terpstra–Kendall (JTK)_CYCLE, a nonparametric method for detecting circadian oscillations in gene expression time-series data [41]. Additional details regarding statistical testing, sample size, and p-values are provided in figure legends. R Studio was used for all statistical analyses and to generate all plots [42].

## Supplementary Material

Supplementary_materials_ddag065
