# Supplementary material for: A patient-derived mouse model reproduces molecular, neurological, and sleep symptoms of SHINE syndrome
Source: Hum Mol Genet. 2026 Aug 7;35(16):ddag065. doi: 10.1093/hmg/ddag065 (PMC13648028; doi:10.1093/hmg/ddag065)
Supplement: Supplementary file 1 [file supplementary_materials_ddag065.zip › Supplemental-Figure-Legends_HMG_ST_07-08-2026.docx]

**SUPPLEMENTAL FIGURE LEGENDS**

**Figure S1**. *Transcriptional regulation of* DLG4. (**A**) ChIP-seq tracks from bHLH transcription factors show binding across the *DLG4* promoter, suggesting the presence of functional E-box motifs (UCSC Genome Browser). (**B**) Predicted locations of E-box (green) and RORE (blue) motifs within the *DLG4* promoter region.

**Figure S2**. *Blots demonstrating* Dlg4*/PSD-95 expression*. (**A**) Gene expression of wild-type and p.(V692Wfs*12) *Dlg4* alleles in the hypothalamus of *Dlg4^+/+^* and *Dlg4^V692Wf*s12/+^* mice using allele-specific PCR (NT, no template control). (**B**) Western blot of PSD-95 protein expression in *Dlg4^+/+^* and *Dlg4^V692Wf*s12/+^* mice, with GAPDH as a loading control. Mouse number is indicated on panels A and B.

**Figure S3**. *Morris water maze*. (**A**) Path efficiency to reach the platform during learning trials (Day 5 acquisition p = 0.008; Day 3 reversal p = 0.011; Day 5 reversal p = 0.028). (**B**) Distance swam to reach the platform during learning trials (p = 0.58). (**C**) Swimming speed during learning trials (p = 0.20). (**D**) Number of entries into the target quadrant during probe trials (*Dlg4^+/+^* males, 3 ± 0.45; *Dlg4^V692Wfs*12/+^* males, 0.5 ± 0.50; p = 0.008; *Dlg4^+/+^* females, 1.6 ± 0.51; *Dlg4^V692Wfs*12/+^* females, 4.2 ± 0.58; p < 0.001). (**E**) Distance traveled during probe trials (p = 0.73). Data represent mean ± SEM from *Dlg4^+/+^* (*n* = 10; 5 males, 5 females) and *Dlg4^V692Wfs*12/+^* (*n* = 8; 3 males, 5 females) mice.

**Figure S4**. *Startle, social choice, and rotarod assays*. (**A**) Pre-pulse inhibition of acoustic startle reflex. (**B**) Proportion of total time spent in each chamber during the social choice assay. (**C**) Preference for the social chamber during the social choice assay (time spent in [social – non-social] / [social + non-social]; p = 0.064). Data represent the mean ± SEM from *Dlg4^+/+^* (*n* = 6; 3 males, 3 females), *Dlg4^V692Wfs*12/+^* (*n* = 6; 2 males, 4 females), and *Dlg4^V692Wfs*12/V692Wfs*12^* (*n* = 6, 3 males, 3 females). (**D**) Latency of 2-month-old (2 mo) mice to fall off the accelerating rotarod (Day 1; *Dlg4^+/+^*, 309.0 ± 15.7 s; *Dlg4^V692Wfs*12/+^*, 250.0 ± 16.4 s; p = 0.029). Data represent the mean ± SEM from *Dlg4^+/+^* (*n* = 10; 5 males, 5 females), *Dlg4^V692Wfs*12/+^* (*n* = 8; 3 males, 5 females). (**E**) Latency of 4-month-old (4 mo) mice to fall off of the accelerating rotarod (p = 0.59). Data represent the mean ± SEM from *Dlg4^+/+^* (*n* = 19; 9 males, 10 females), *Dlg4^V692Wfs*12/+^* (*n* = 18; 8 males, 10 females). (**F**) Latency of 6-month-old (6 mo) mice to fall off of the accelerating rotarod (p = 0.33). Data represent the mean ± SEM from *Dlg4^+/+^* unrelated (UR; *n* = 7; 4 males, 3 females), *Dlg4^+/+^* littermates (*n* = 8; 4 males, 4 females), *Dlg4^V692Wfs*12/+^* (*n* = 8; 4 males, 4 females), and *Dlg4^V692Wfs*12/V692Wfs*12^* (*n* = 8, 4 males, 4 females).

**Figure S5**. *Wheel running and patient actigraphy*. (**A**) Wheel running activity traces. Solid lines represent the mean; shaded areas indicate ± SEM. Sample sizes are detailed above. (**B**) Double-plotted actigraph of patient activity and sleep. The patient was 10 years old during the data collection period. Yellow boxes highlight hyperactivity in male mice and the patient in the hours preceding the resting phase. (**C**) Lomb-Scargle periodogram of patient activity (p < 0.05).

**Figure S6**. *PiezoSleep assay*. (**A**) Histogram of counts of sleep bouts for increasing sleep bout lengths up to 75 minutes in male mice in LD. (**B**) Phase shift of activity (relative to Day 1; 6hr Adv, 6-hour phase advance; 6hr Del, 6-hour phase delay). Cosinor analysis of hourly wake probability was performed for each mouse to estimate daily acrophase. Solid lines represent the mean; shaded areas indicate ± SEM. (**C**) Entrainment rate to 6-hour phase advance and 6-hour phase delay (p = 0.854). Sample sizes are detailed above.
